# Design and Analysis of a Pitch Fatigue Detection System for Adaptive Baseball Learning

**DOI:** 10.3389/fpsyg.2021.741805

**Published:** 2021-12-13

**Authors:** Yi-Wei Ma, Jiann-Liang Chen, Chia-Chi Hsu, Ying-Hsun Lai

**Affiliations:** ^1^Department of Electrical Engineering, National Taiwan University of Science and Technology, Taipei, Taiwan; ^2^Department of Computer Science and Information Engineering, National Taitung University, Taitung, Taiwan

**Keywords:** adaptive baseball learning, pitch fatigue detection, computer visions, smart sports, machine learning

## Abstract

Owing to the rapid development of information and communication technologies, such as the Internet of Things, artificial intelligence, and computer vision, in recent years, the concept of smart sports has been proposed. A pitch fatigue detection method that includes acquisition, analysis, quantification, aggregation, learning, and public layers for adaptive baseball learning is proposed herein. The learning determines the fatigue index of the pitcher based on the angle of the pitcher's elbow and back as the number of pitches increases. The coach uses this auxiliary information to avoid baseball injuries during baseball learning. Results show a test accuracy rate of 89.1%, indicating that the proposed method effectively provides reference information for adaptive baseball learning.

## 1. Introduction

The sports industry offers considerable economic benefits. The use of emerging technologies in the sports industry promotes cross-field cooperation (Liu et al., 2017; Zhang et al., 2018). The integration of sports and information technology supports “smart sports,” which involves the intelligent use of scientific information pertaining to athletes. Smart sports use numerous sensors to acquire information regarding the behavior of athletes, allowing sports-related data to be analyzed to enhance competitive performance (Gathercole et al., 2015; Blanchard et al., 2019; Buric et al., 2019). In recent years, governments have actively promoted the integration of sports and information and communication technology (ICT), including the use of scientific methods and information technology to identify the movements and postures of athletes to improve their performances and physical fitness, as well as to prevent injury. Herein, the current technological development in baseball as a smart sport is discussed. Because ICT is used to acquire baseball-related data, which are analyzed *via* computer vision, relevant data are no longer merely statistics. In fact, they can be used to analyze offenses and defenses intelligently in the baseball field (Chakraborty and Meher, 2013; Shih, 2017).

In a baseball game, any decision, such as the scheduling of players, can affect the result. Therefore, team experts typically discuss various events that affect performance on the court. Subsequently, they generate statistics to use as references while making decisions when they encounter similar situations in the future, and thus increase their probability of winning a game. However, the accuracy of decision-making can still be further improved using statistical information and expert judgment (Phillips, 2015; O'Sullivan et al., 2018). This study focused on the “appropriate time for pitcher change.” The pitcher critically affects the results of a baseball game. When a pitcher does not perform well, the defensive coach will consider replacing him. However, one of the key judgments is the sports fatigue of pitcher. Sports fatigue not only often affects the results of the game, but also energy feeling may also cause sports injuries during normal practice (Harwood et al., 2015). Boolani et al. (2019) present supporting evidence for the view that fatigue perception and energy are independent. This study proposes an assumption that energy and fatigue are different perception states, which should be measured with a separate unipolar scale, and tailored therapies should be used to treat fatigue. Studies have shown that the mechanisms of driving energy and fatigue changes are likely to be different. Therefore, many studies have also tried to measure different mental and physiological fatigue based on different information, such as heightened and deliberate concentration, intense effort, sensors, and ECG (O'Connor and Puetz, 2005; Swann et al., 2017; Guaitolini et al., 2020; Luo, 2021).

In order to effectively detect sports fatigue and establish adaptive learning, the objectives of the study are listed below:

1. Computer vision will be effectively used in smart sports.
  With the rapid development of artificial intelligence technology, there are more and more cases of establishing smart sports analysis through computing vision. Zhang et al. (2018) use of motion sensor and camera to determine the tennis serve action. Arai et al. (2019) and Liu et al. (2020) also proposed an athlete training system based on sports vision. This study uses image recognition to find out the motion of baseball players' limbs and then analyzes sports fatigue of players.
2. Body movements will be analyzed for sports fatigue.
  With sports fatigue, the body movements of athletes will also show different changes. Therefore, this study attempts to detect whether athletes are fatigued based on their body movements. The research results echo that Carroll and Hansen (2019) and Clemente et al. (2021) find out sports fatigue according to the different body or muscle changes. However, it is different than studies of Carroll and Hansen (2019) and Zhang et al. (2018) combined with motion sensors to help detect athletes' movements. This research establishes the elbow valgus and trunk flexion angle as key features and then combines interval time of pitching to detect sports fatigue. In addition, Burdack et al. (2020) also used pattern recognition of the sports correlation between time and fatigue changes. The research focuses on the use of Support Vector Machine (SVM) for the discussion and analysis of whether the fatigue characteristics of the exercise mode can be observed. The experimental results also echo that fatigue characteristics correspond to changes in body movements. Subsequently, it compares this sum with a T-value to calculate the fatigue point.
3. Adaptive baseball learning system will be adapted to improve sports fatigue.
  Excessive sports fatigue will have a higher risk of sports injuries with energy feeling. According to the experimental investigation, this study proposes that the adaptive learning system will detect athlete fatigue more effectively than traditional fixed training or observing the results of pitching. The system will actively remind the pitcher when it detects different physical movements or fatigue.

## 2. Literature Review

### 2.1. Sports Fatigue

Every athlete experiences fatigue, which reduces their strength, alters their posture, and affects their physical and mental performances (Bishop, 2012). In addition, fatigue affects a person's behavioral, emotional, and spiritual disposition. A global report stated that approximately 20% of adults claim to have experienced fatigue at any specified time. It is crucial to know the causes of fatigue in athletes to prevent or solve problems. Athletes who play while fatigued may suffer from sports injuries (Irawan and Long-Ren, 2019). The team manager and coach are responsible for protecting players from injury. Typically, sports protectors are recruited to train players for strength, muscular endurance, and physical fitness to reduce the probability of fatigued players during a game (Benson et al., 2013; Coutinho et al., 2018). Sports protectors support not only the health and careers of players but also the victory of the team. Player fatigue affects team performance and can cause teams to lose their games.

Schiphof-Godart et al. (2018) uses multiple perspectives to explore how mental fatigue interacts with motivation and performance in endurance sports, and guides athletes' sports behavior and performance effectiveness. The research focuses on the relationship between mental fatigue and performance. Burdack et al. (2020) propose pattern recognition of the correlation between time and fatigue changes. The research focuses on two issues: (1) whether the fatigue characteristics of the exercise mode can be observed. (2) when fatigue increases, will personal exercise patterns change. The research focuses on the use of Support Vector Machine for the discussion and analysis of the above problems. Some study focus on the impact of mental fatigue on the effectiveness of sports behaviors. The purpose is to compare the effects of mental fatigue and control conditions on the exercise performance of small-sided games (Badin et al., 2016; Clemente et al., 2021).

### 2.2. Fatigue Definition and Detection Methods

The performance of a baseball player is determined not only by their ability but also by their physical condition (Keeley et al., 2014). The performances of pitchers, hitters, and fielders affect the results of a baseball game. Pitcher fatigue is typically reflected in changes in posture, ball velocity, and time between pitches (Carroll and Hansen, 2019). A coach who fails immediately to notice pitcher fatigue may contribute not only to the loss of a game but also to a possible injury to the pitcher, which may affect their career. Therefore, the detection of pitcher fatigue critically affects the results of a game (Magnusson et al., 1994; Birfer et al., 2019).

Carroll and Hansen (2019) defined fatigue as an exercise-induced loss of muscle force-generating capacity. Fatigue reduces strength and joint stability, thereby affecting postural control, which results in inaccurate control by a player during his movements. Loy et al. (2018) propose that energy and fatigue are two individual and separate but related psychological states, rather than opposites on a single bipolar continuum. Energy is defined as an individual's potential to perform mental and physical activity. It is usually called fatigue when people are exhausted, sluggish, weary, tired, or feeling worn out. Kowalski et al. (2021) find that trait physical and mental fatigue and energy can be predicted with postural control and gait. Mahoney et al. (2021) uses exploratory research to show that energy and fatigue are differently related to gait characteristics and balance. They examine the relationship between self-reported fatigue, energy, gait, and balance characteristics of healthy in young people. The research results show that the feeling of fatigue and energy has a unique impact on the gait characteristics and balance of healthy in young people.

For baseball, the body posture of a pitcher may change during a game and hence affect the game results or their performance. Therefore, accurate identification of fatigued players, followed by their replacement in a timely manner, is critical in a game (Yang et al., 2016). Lizzio et al. (2020) demonstrated that pitcher fatigue is associated with several factors, such as changes in the pitching posture, increased intervals between pitches, changes in attitude, frequent sweating, changes in ball velocity, and unstable ball control (Natwa et al., 2018).

In this study, pitcher fatigue was detected based on three methods, i.e., changes in the elbow valgus angle, trunk flexion angle, and time between pitches. The fatigue degree of a pitcher was calculated based on video images of a game. The images were used to identify changes in the pitcher's joint angles and to calculate the time between pitches; a pitcher fatigue value was determined based on the three above mentioned methods for each pitch (Okoroha et al., 2018).

## 3. Proposes Adaptive Baseball Learning System

### 3.1. Assumptions

To eliminate force majeure factors and to reduce the computational complexity of the proposed system in the experiment, the following assumptions are made.

1. Any difference in strength between the pitcher and batter is negligible.
  This study focuses primarily on the fatigue analysis of a pitcher's pitching posture, followed by its verification based on a fatigued batter hitting the ball. Therefore, the relationship between the strength difference between the pitcher and batter is preliminarily excluded.
2. The pitcher's posture does not change with the pitch type.
  In baseball pitching, many different pitching types exist, such as finger balls, slippery balls, and straight balls. Although the different pitching types will affect the pitcher's posture, the pitcher will attempt to maintain the same pitching posture to prevent the batter from assessing the pitching type. We initially assumed that the pitcher does not change their pitching posture based on the pitching type.
3. The pitcher is not injured during the game.
  When a pitcher is injured, they assume a different pitching posture. In this study, it is preliminarily assumed that no pitcher is injured during practice or competition.

### 3.2. Design of Quantification and Aggregation Learning Architecture

The use of quantification and aggregation learning architecture (QALA), which includes a collection layer, an analysis layer, a quantification layer, an aggregation layer, a learning layer, and a public layer, is proposed herein. The collection layer is primarily used to acquire images from cameras and information from sensors. Cameras were used to capture a video stream from which the posture of the pitcher was obtained. Meanwhile, sensor devices were used to acquire the sensing signal, from which the pitcher's pitching speed and angles were obtained. The analysis layer was primarily used to determine the pitcher's posture and pitch interval from the video stream, where the elbow valgus angle, trunk flexion angle, and time between pitches were extracted.

The quantification layer was primarily used for the calculation and quantification of fatigue, which involve the evaluation of angular and time changes. The aggregation layer was primarily used for the aggregation of various fatigue quantitative data, which includes the calculation of fatigue points and the assessment of the accuracy of the validation data. The learning layer was primarily used for fatigue model training, data verification, and result display; it involves the training, validation, and testing of modules. The public layer was primarily used to store relevant information in a player information database and player prediction record database.

### 3.3. Analysis Methods of Data Features

In this study, image analysis and learning perspectives were employed to enable a team to understand the fatigue condition of the pitcher more effectively. The data features were analyzed *via* the following three methods to identify a fatigued pitcher.

- Extract the elbow valgus angle: This method was used to obtain the elbow valgus angle. OpenPose software was used to denote joint points in an image, provide the coordinates of those points, and then calculate the elbow valgus angle from the coordinates of particular joints.
- Extract the trunk flexion angle: This method was used to obtain the trunk flexion angle. OpenPose software was used to denote joint points in an image, provide the coordinates of those joints, and then calculate the trunk flexion angle from the coordinates of particular joints.
- Extract the time between pitches: This method was used to obtain the interval between pitches.

**Figure 1:**
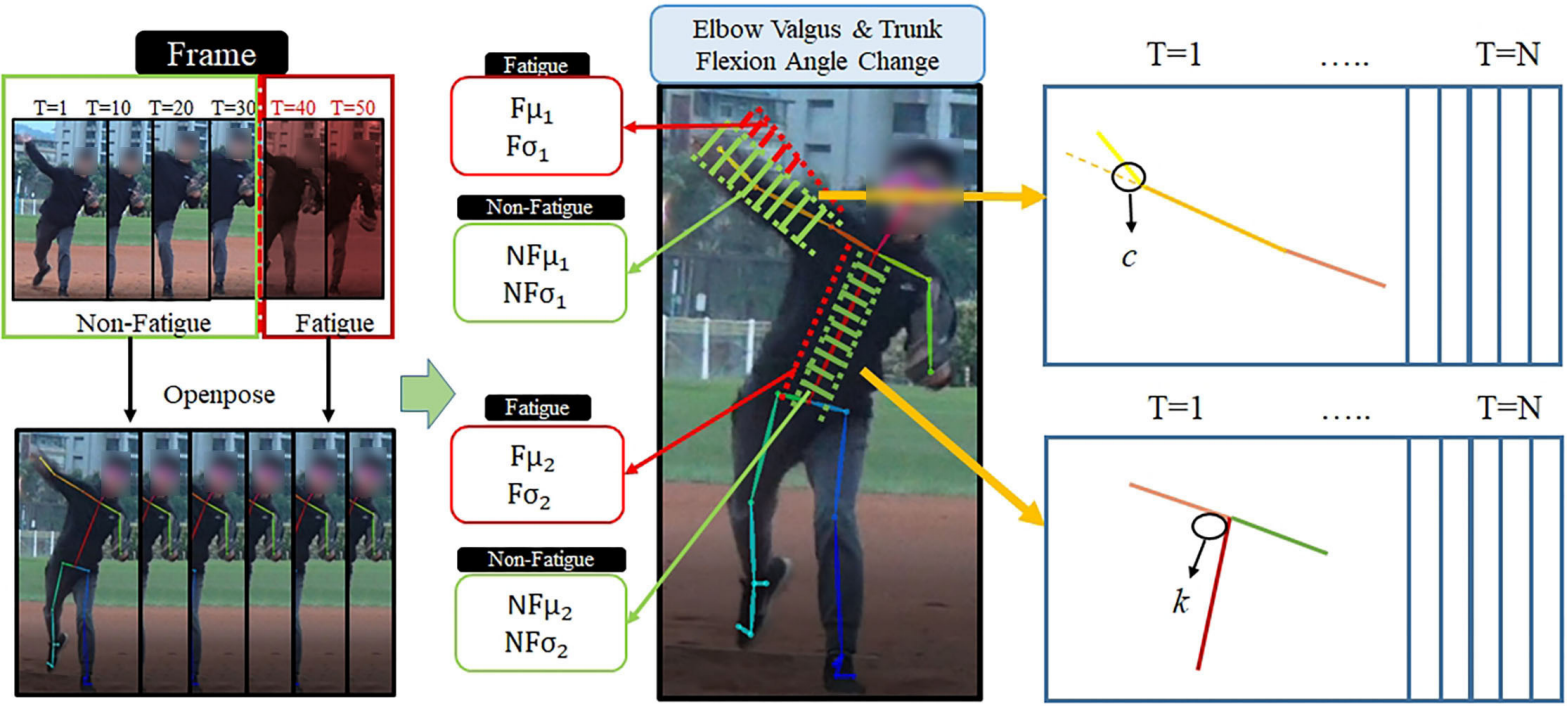
Elbow valgus and trunk flexion angle analysis method.

The first two methods capture pitching images from a baseball game video to determine the changes in the pitcher's elbow and trunk flexion angles, as shown in Figure 1. Images are captured when the ball is at its highest point during the pitch. OpenPose is used to locate the pitcher's joint in each picture; subsequently, the coordinates are used to calculate the joint angle. The many characteristics of pitcher fatigue include low ball velocity, frequent slight movements, posture changes, and unstable pitching. When a pitcher pitches while fatigued, the probability of shoulder or elbow injury increases. Pitching under excessive fatigue may cause permanent arm injuries that cannot be healed, thereby affecting the pitcher's career. A coach who can identify a fatigued pitcher can not only reduce the number of points lost and increase the probability of winning games but also protect pitchers from injury. In this study, image analysis and learning perspectives were used to determine the fatigue state of a pitcher. The proposed system uses three methods to determine the fatigue state of a pitcher.

The change in the elbow angle of the pitcher is an important basis for identifying pitcher fatigue. A pitcher's performance is adversely affected by his physical fatigue, which can manifest as instability in the elbow angle. When a pitcher is not tired, the pitching arm appears horizontal when the ball is thrown, and the elbow angle is small. When a pitcher feels tired, the pitching arm is raised considerably when the ball is thrown, leading to a large elbow angle. However, a coach cannot visually determine changes in the angle of the pitcher's arm.

The change in the trunk flexion angle is another basis for identifying pitcher fatigue. When the body is not tired, the trunk flexion can often be pressed lower than the typical state during a pitch, thereby completing the pitching action. This results in a large angle between the shoulder and the back. When the body is tired, the trunk flexion cannot be pressed lower than the typical state during a pitch, and the pitching action is incomplete. This results in a small angle between the shoulder and the back. A coach cannot visually determine changes in the angle of the pitcher's shoulder or back. Therefore, an image recognition technique must be used to identify any slight changes in the flexion angle of the pitcher to assess fatigue.

The time between pitches is another basis for assessing pitcher fatigue. When a pitcher is tired, both the angles of the body joints and the time between pitches change. When a pitcher is tired, the time between pitches is long because the pitcher paces back and forth or decelerates and rests for a longer duration.

### 3.4. Information Quantification and Aggregation

After the information is collected and analyzed using three methods, variations of angles and intervals must be converted to fatigue quantitative data. The elbow valgus angle (c) means that the elbow angle of the pitcher's arm when throwing the ball. The trunk flexion angle (k) means that the trunk flexion angle of the pitcher's body when throwing the ball. When the pitcher is not tired, the trunk flexion angle can often be pressed lower than usual when pitched and the pitching action is made completely. The time between pitches (t) is defined as time from the pitcher's pitch to the next pitch. When the pitcher is tired, the time between the pitch is long. The reason is that the pitcher will pace back and forth or slow down the pitch, and get more rest time.

In the stage of quantifying fatigue, the total numbers of balls pitched are distinguished with fatigue and non-fatigue interval according to the fatigue interval index α. For example, when the total number of balls pitched is 100, and α is 0.6, it means that the first 60 balls are in the non-fatigue interval and the next 40 balls are in the fatigue interval. The average and SD of the features between the non-fatigue interval (*x*_*m*_) and fatigue interval (*y*_*m*_) were calculated.


(1) $$\begin{array}{l} x_{m}=NF_{um}+NF_{\sigma m},m=1,2,3 \end{array}$$

(2) $$\begin{array}{l} y_{m}=F_{um}+F_{\sigma m},m=1,2,3 \end{array}$$

*NF*_*um*_ is the average of the values in the non-fatigue interval. *NF*_σ*m*_ is the standard deviation of the values in the non-fatigue interval. *F*_*um*_ is the average of the values in the fatigue interval. *F*_σ*m*_ is the SD in the fatigue interval, and *m* is the number of the three data feature methods included: elbow valgus angle, the trunk flexion angle, and the time between pitches. The intersection of the fatigue interval and the non-fatigue interval is used as a range for quantifying fatigue. After the fatigue value is obtained using the above three methods, fatigue values *A*_1_, *A*_2_, and *A*_3_, respectively, are obtained.


(3) $$\begin{array}{l} A1=\{\begin{array}{c} \;0,\text{if }c<x_{1}\\ \;\frac{c-x_{1}}{y_{1}-x_{1}},\text{if }y_{1}\geq c\geq x_{1}\\ \;1,\text{if }c>y_{1} \end{array} \end{array}$$

(4) $$\begin{array}{l} A2=\{\begin{array}{c} \;0,\text{if }k>x_{2}\\ \;\frac{x_{2}-k}{x_{2}-y_{2}},\text{if }x_{2}\geq k\geq y_{2}\\ \;1,\text{if }k<y_{2} \end{array} \end{array}$$

(5) $$\begin{array}{l} A3=\{\begin{array}{c} \;0,\text{if }t<x_{3}\\ \;\frac{t-x_{3}}{y_{3}-x_{3}},\text{if }y_{3}\geq c\geq x_{3}\\ \;1,\text{if }t>y_{3} \end{array} \end{array}$$

After quantifying fatigue value through three different methods, the fatigue values *A*_1_, *A*_2_, and *A*_3_ of elbow valgus angle, trunk flexion angle, and time between pitch are obtained, respectively. The sum of the three fatigue values is an important reference for assessing a pitcher's fatigue. The fatigue values are summed to yield *M*_*total*_ as the total fatigue index.


(6) $$\begin{array}{l} M_{total}=A_{m}*W_{m} \end{array}$$

The weights *W*_*m*_ are defined in terms of variations of elbow valgus angle, trunk flexion angle, and time between pitches. *T* value is defined as a fatigue threshold. If *M*_*total*_ exceeds *T*, then it is the number of balls pitched when the pitcher is tired.

### 3.5. Learning Methods

**Figure 2:**
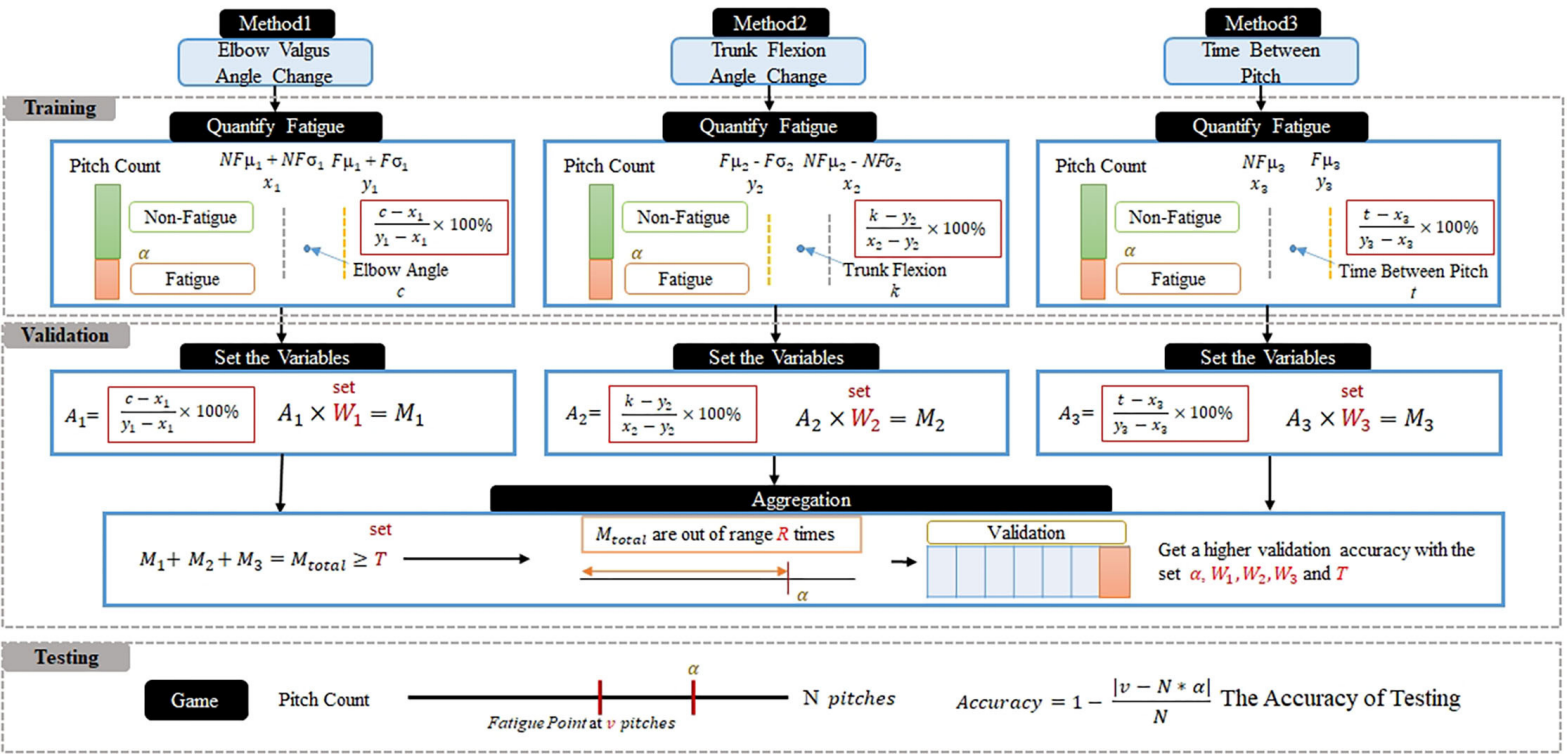
Data learning and testing flow.

A learning framework comprising three stages, i.e., training, validation, and testing, are proposed herein. The framework was used to systematically learn the fatigue data of pitchers and to learn a model that can accurately identify pitcher fatigue. In the training stage, the three proposed analysis methods are applied to obtain quantitative information from the image data acquired. Accordingly, the behavioral characteristics of the pitcher that are associated with fatigue and non-fatigue can be identified. In the validation stage, a significant amount of game data is used to determine the accuracy of the parameter configuration and to identify the best parameter settings for the validation dataset. In the testing stage, a verified parameter configuration and actual game data are used to verify the accuracy of the testing data used in the actual system operation, as shown in Figure 2.

The working stages were the offline and online stages. This section focuses on the flow of the system at each stage. The offline stage includes the training and validation phases. In the training phase, the system receives historical data from the video games. Three features were extracted from the videos to evaluate the fatigue values of the pitcher. The mean and SD of the non-fatigue and fatigue intervals were calculated. Fatigue was quantified using the mean and SD. Finally, the player's information was stored in a team database. In the validation phase, the system uses the fatigue value stored in the database during the training phase. The parameters were set, and cross-validation was performed to create a reliable model. The validation accuracy was calculated using the fatigue point and set parameters. Finally, the most suitable parameters were selected and used to construct a pitcher-fatigue prediction model using Support Vector Machine (SVM) algorithms in online stages.

## 4. Experiments and Analysis

The performance of the proposed system was analyzed. The competition images acquired were tested using the proposed method. The relationship between the parameter settings and the results was analyzed. Experimental data pertaining to the games from the CPBL 2018 Fubon Titans baseball team, with pitcher Loree, were used. A public video of the baseball game was obtained from the Internet. Seven games involving Loree as the pitcher were selected at random for seven-fold cross-validation, and the testing data were obtain from one game. The fatigue values were obtain using the three methods for each pitch in each game and then added to obtained the *M*_*total*_. The system calculates the number of balls, *R*, when *M*_*total*_ ≥ *T* before α, and then performs cross-validation. The fatigue point (*F*_*P*_) is calculated in the previous sessions for verification.

(7) $$\begin{array}{l} F_{P}=\frac{1}{n-1}\overset{n-1}{\underset{i=1}{\sum }}R_{i} \end{array}$$

Then, the accuracy of the fatigue point (*A*_*f*_) is calculated with the distance of the original defined target α.


(8) $$\begin{array}{l} A_{f}=1-\frac{\left|F_{p}-N_{i}*\alpha \right|}{N_{i}} \end{array}$$

Finally, the final accuracy rate is obtained by averaging the accuracy rate of the cross-validation.

### 4.1. Detection Accuracy With Different Parameters

The goal of the offline phase is to identify a suitable parameter for the proposed system. Parameters α, *W*_1_, *W*_2_, *W*_3_, and *T* were set to various values, and their effects on accuracy were investigated. The parameter settings that yielded an effective prediction model were identified. Eight cases involving training data were considered experimentally.

**Figure 3:**
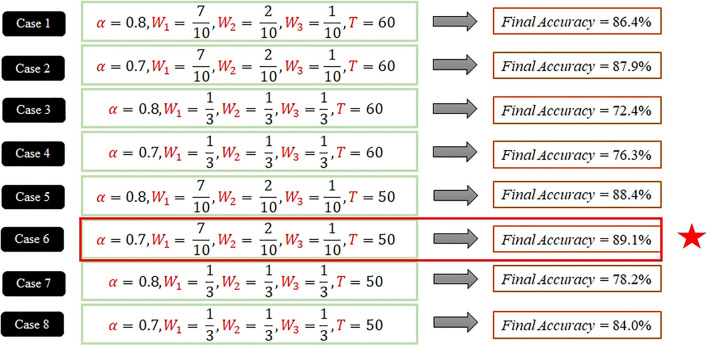
Cross-validation of eight cases.

For Case 1, under settings α = 0.8, *W*_1_ = 0.7, *W*_2_ = 0.2, *W*_3_ = 0.1, and *T* = 60, the fatigue point was calculated as the total number of pitches multiplied by α = 0.8. The first field was regarded as the validation set. The average fatigue point, *R*_*n*_, of the second to seventh games was 4.7. The average fatigue point was used in the validation set of the first game to obtain the 45th pitch, which was set as the fatigue point. Next, it was subtracted from the 74th pitch with a preset fatigue point α = 0.8. The accuracy rate was calculated using formula 8. After obtaining seven sets of different validation accuracies, the system outputs the average value for the final accuracy of this case, i.e., 86.4%. Similarly, the final correct rate for the other cases were obtained using this method as demonstrated in Figure 3.

For case comparison, α = 0.7 gets the better the accuracy rates than α = 0.8 when *W*_1_, *W*_2_, *W*_3_, and *T* are fixed. Using *W*_1_, *W*_2_, *W*_3_ as variables, the accuracy rate of *W*_1_ = 0.7, *W*_2_ = 0.2, and *W*_3_ = 0.1 is greater than the accuracy rate of *W*_1_ = 0.33, *W*_2_ = 0.33, and *W*_3_ = 0.33. Comparing the accuracy rate between *T* = 60 and *T* = 50, when α, *W*_1_, *W*_2_, and *W*_3_ are the same setting, the accuracy rate of *T* = 50 is greater than the accuracy rate of *T* = 60. Before eight cases, the system compared the cases with pair and found the setting α = 0.7, *W*_1_ = 0.7, *W*_2_ = 0.2, *W*_3_ = 0.1, and *T* = 50 yielded the highest system accuracy rate of 89.1%.

### 4.2. Adaptive Learning Experience

Based on online testing, α = 0.7, *W*_1_ = 0.7, *W*_2_ = 0.2, *W*_3_ = 0.1, and *T* = 50 and a fatigue point of 6 are set as parameters. In this study, members of college baseball clubs were invited to conduct experimental tests. In order to avoid affecting the club practice, we conducted experimental tests 1 day in the club's practice time. Finally, 30 baseball players were recruited to participate in a learning experiment. The subjects were 18–24 years old. They were randomly segregated into experimental and control groups. After a preliminary warm-up exercise, a pre-test survey of fatigue was conducted. The experimental group used the proposed dynamic pitching fatigue detection to assist in their judgment, whereas the control group depended only on the coach for their assessment. After 2 h of training, a post-exhaustion survey was conducted. At this time, covariate analysis was used to perform a fatigue verification analysis. Analysis of Covariance (ANCOVA) is a statistical control approach that uses statistical methods to eliminate errors that may affect the accuracy of an experiment. In this study, the ANCOVA was based on the difference between the control and experimental groups at the beginning of the pre-test and in the post-test, obtained through regression and adjusted to equivalent scores to confirm that the dependent variable scores can be based on the same scores to achieve more accurate analysis results is shown in Table 1.

**Table 1:** Summary of descriptive statistics analysis.

**Groups**	***N***	**Pre-test**		**Post-test**		**Adjusted mean (M')**
		**M**	**SD**	**M**	**SD**	
	15	56.27	5.42	77.20	4.62	77.94
	15	59.47	5.14	74.27	4.76	73.53

The results show that the QALA system used in this study effectively detected and prevented sports injuries caused by excessive fatigue during baseball training. Between the two groups, the mean of fatigue scores were 56.27 and 59.47 in the pre-test. The mean of fatigue scores were 77.20 and 74.27 in the post-test. After covariation, the post-test scores were adjusted to 77.94 and 73.53.

For the hypothesis pertaining to the homogeneity of the regression coefficients within the group, the *p*-value was 0.463, which indicates that the homogeneity of the regression coefficients can be analyzed for covariate results shown in Table 2. In the covariate analysis, *F*-value was 8.018 and *p*-value was 0.009. The results show that the adaptive learning system effectively detected and prevented sports injuries caused by excessive fatigue during baseball learning and gaming.

**Table 2:** Summary table of analysis of covariance (ANCOVA).

**Tests of between-subjects effects**							
**Dependent Variable: a test**							
**Source**	**Sum of squares**	**df**	**Mean square**	**F**	**Sig**.	**Partial eta squared**	**Observed power**
Corrected model	231.777	2	115.889	6.983	0.004	0.341	0.895
Intercept	554.301	1	554.301	33.400	0.000	0.553	1.000
ptest	167.244	1	167.244	10.077	0.004	0.272	0.864
Sets	133.065	1	133.065	8.018	0.009	0.229	0.779
Error	448.089	27	16.596				
Total	172746.000	30					
Corrected total	679.867	29					

*R squared = 0.341 (adjusted R squared = 0.292). Computed using alpha = 0.05*.

## 5. Discussions

### 5.1. Limitations

The experimental samples are tested between 18 and 24 years old and the experimental process is tested in a standard baseball practice field. Whether, the analysis method is still applicable to other ages or non-standard baseball fields is still to be studied. The study captures changes in the pitcher's body angle with a questionnaire for fatigue surveys. However, this method explores how pitchers feel about self-fatigue. There is still not enough evidence to clearly distinguish the measurement and judgment of energy and fatigue by O'Connor and Boolani findings (O'Connor and Puetz, 2005; Boolani et al., 2019). They assumed that energy and fatigue are different perception states, which should be measured with a separate unipolar scale, and tailored therapies should be used to treat fatigue.

Finally, the ANCOVA method is adopted to avoid the difference in basic fatigue cognition between the two groups (Tsay, 2004; Salvador et al., 2009). However, it is still impossible to completely rule out the responsiveness of some personnel to the system. This study is mainly for results of sports rehabilitation analysis of strenuous exercise and cannot be broadly deduced to sports rehabilitation analysis.

### 5.2. Summary of Contributions and Findings

This study proposes that the adaptive learning system called QALA enables the system to learn a relatively effective prediction model to determine fatigue in pitchers. The proposed methods include analyzing the elbow valgus angle, trunk flexion angle, and time between pitches. A fatigue quantification method based on variations in the elbow valgus angle, where the trunk flexion angle transforms physical features into numerical values, is proposed herein. It uses images and pitching times to identify a fatigued pitcher during a game, thereby enabling the coach to make more accurate decisions about when to replace the pitcher. Accordingly, better tactical decisions can be made and the pitchers can be protected. Furthermore, detecting changes in the posture of a pitcher *via* image analysis in a timely manner can support early tactical decisions pertaining to pitcher replacement and increase the probability of winning a game.

In experience, seven games were used in seven-fold cross-validation to generate a relatively high accuracy rate when the parameters were set by the system, and a validation accuracy of 89.1% was achieved. In the adaptive learning experiment, the investigation results show that the proposed method is indeed effective for the pitcher to detect fatigue during practice. However, the effect size value is 0.229. This shows that the effect of this method on fatigue perception is still small. In the qualitative interviews, most subjects feel that this system can reduce the incidence of sports injuries. But there are still some subjects who believe that they have not yet reached a very tiring situation. Therefore, this system would detect fatigue, it still has different cognitions for different people for fatigue alarm. This shows that the degree of fatigue for different people's support exercises and even competitions still needs follow-up research. In the future, this research will try to combine the facial change information or other image information to strengthen the detection methods for sports fatigue.

## Data Availability Statement

The original contributions presented in the study are included in the article/supplementary material, further inquiries can be directed to the corresponding author/s.

## Author Contributions

Y-WM proposed the whole research theory and literature research. J-LC designed the feature extraction method of pitching data. C-CH collected the experimental data and programmed the system code. Y-HL designed the pre-test and post-test flow of adaptive baseball learning and analyzed the results. All authors contributed to the article and approved the submitted version.

## Conflict of Interest

The authors declare that the research was conducted in the absence of any commercial or financial relationships that could be construed as a potential conflict of interest.

## Publisher's Note

All claims expressed in this article are solely those of the authors and do not necessarily represent those of their affiliated organizations, or those of the publisher, the editors and the reviewers. Any product that may be evaluated in this article, or claim that may be made by its manufacturer, is not guaranteed or endorsed by the publisher.
